# Two-stage optimization scheme of routing scheduling from a single distribution center to multiple customers

**DOI:** 10.1007/s12351-023-00747-z

**Published:** 2023-03-20

**Authors:** Matías Núñez-Muñoz, Rodrigo Linfati, John Willmer Escobar

**Affiliations:** 1 School of Industrial Engineering, Universidad del Bío-Bío, 4030000 Concepción, Chile; 2 Department of Industrial Engineering, Universidad del Bío-Bío, 4030000 Concepción, Chile; 3 Department of Accounting and Finance, Universidad del Valle, 760001 Cali, Colombia

**Keywords:** Logistic, Assignment model, Scheduling, Data processing

## Abstract

This paper proposes a solution scheme for the problem of scheduling deliveries from distribution centers. The solution scheme is developed in two phases. The first phase corresponds to the collection and processing of data, and the second phase includes the design of a customer assignment model based on the data of the first phase. The first phase provides the characteristics of the customers, the number of orders to be dispatched, and the number of visited customers. In the second phase, optimization techniques are used, considering scheduling and assignment problem-solving methodologies. The optimization model provides the correct assignment of customer orders to vehicles, reducing the number of trips and the fleet's size and occupation percentage. The proposed solution scheme is developed with a company operating a distribution center responsible for dispatching vehicles to all retail customers, wholesalers, and distributors in the Province of Concepción, Province of Arauco, and Province of Itata in Chile. The obtained results are prominent and show that the proposed methodology can be extended to companies performing storage, transportation, and distribution tasks with similar characteristics.

**Supplementary Information:**

The online version contains supplementary material available at 10.1007/s12351-023-00747-z.

## Introduction

The decision-making process in logistics generates uncertainty about the future effects associated with processes related to supply chain planning. Supply chain decisions need quantitative support so that the probability of success is greater than the current situation. In recent years, the tools for improving decision-making have diversified. Statistical instruments and the field of data science are fundamental support for business decision-making. Terms such as "data analysis," "data mining," and "big data" are increasingly common. Another prominent methodology for problem-solving and decision-making is formulating problems through optimization models. This tool solves problems with many variables and factors that are practically impossible to solve manually. This work applies a combination of tools for the land transport industry (optimization and data mining) to improve logistics decisions and last-mile management.

The transportation industry has significantly changed and grown recently, mainly attributed to the evolution of eCommerce. Monroy (2020) comments that the growth of eCommerce during 2020 came with significant challenges and a different way of addressing transportation processes. Currently, the scheduling of deliveries and the logistics processes of product dispatch does not, for the most part, have quantitative support (Branke and Pickardt 2011).

A route calendar is constructed where the destination areas and the day on which the route will be taken are specified (Bunte and Kliewer 2009). This scheduling is usually established and reassessed annually considering two objectives: (1) the conditions of the service agreement with the customers and their influence on the quality and satisfaction with the service, and (2) prioritizing visits to destination nodes that are located in an area indicated in the calendar. In this work, a weekly planning horizon is considered, with some specific cases in which a visit frequency equal to or greater than weekly is optional.

This paper proposes a solution scheme for the problem of deliveries for a Distribution Center by using quantitative techniques and tactical-strategic methodologies to deal with dispatch logistics operations. The proposed methodology aims to reduce the deficiencies and limitations in the planning and scheduling of deliveries, minimize the uncertainty regarding efficient and effective results from the processes, and increase the quality and level of service delivered to the customer. In this case, a company with an average of 19.25% of shipments not delivered on time is considered.

Section 2 specifies the literature related to the problem of vehicle scheduling for distribution centers and its associated decisions. Section 3 describes in detail the proposed methodology for the problem under consideration. Finally, in Sect. 4, the conclusions of the obtained findings are detailed, and future works are proposed.

## Literature review

A supply chain is formed by diverse echelons directly or indirectly involved in satisfying a customer request, maximizing the total value generated (Chopra et al. 2013). One of the most important decisions within supply chain planning involves planning transport logistics processes that link with the final customer, which is called last-mile logistics. The last-mile logistics refers to the final section of the goods delivery process. It is the most expensive and problematic area of the supply chain, where the main complication is product delivery (Juhász and Bányai 2018). Generally, diverse factors and high logistic costs are incurred with the delivery process of the companies (Juhász and Bányai 2018). However, significant benefits of the solutions provided by the last-mile logistics could also be identified, such as time savings, safety, energy savings and lower environmental impacts, level of customer service, mobility, and productivity.

Several early published works dealing with scheduling decisions have considered the combination of routing and scheduling problems within real environments. For example, Li et al. (2008) considered a truck scheduling problem for solid waste collection in Porto Alegre, Brazil. This problem is similar to our problem, where "good" daily truck schedules can be designed on a set of previously defined collection trips. The objective of this work is to minimize the total operating and fixed costs of the trucks. A heuristic approach was proposed incorporating an auction algorithm and a dynamic penalty method to solve this problem. Unlike the work proposed by Li et al. (2008), we solve a real routing scheduling problem by finding the optimal solution for a large-scale case. Dror and Trudeau (1996) study an optimization problem in money flow delivery planning. The routing and inventory problem is analyzed from the perspective of deterministic and stochastic customer demand. Similarly, Gayialis and Tatsiopoulos (2004) proposed a decision support system for the route scheduling problem in an oil production company. The objectives of the new tool are the optimal use of the resources of the distribution network, the reduction of transportation costs, and the improvement of customer service.

Another real interesting problem considering the problem of waste collection activities in Hanoi, Vietnam, was studied by Tung and Pinnoi (2000). The problem was formulated using a mixed-integer programming model, and a two-phase heuristic procedure have proposed. Ghanim and Mamaghani (2012) considered the problem of planning vehicle schedules from depots. In particular, different time windows were considered for different products, load constraints, multiple trips, and an additional depot for some products. Similarly, Day et al. (2009) considered the problem of cyclical inventory replenishment for a regional DC of a company that supplies, distributes, and manages carbon dioxide (CO2) inventory in more than 900 customer sites in Indiana. The authors propose a three-phase heuristic for each DC's cyclic inventory routing problem. Finally, Mathlouthi et al. (2021) addressed a technician routing and scheduling problem motivated by an application for repairing and maintaining electronic transactions equipment. A metaheuristic algorithm based on tabu search, coupled with an adaptive memory, is proposed.

Several authors proposed variants of the well-known Single Depot Vehicle Scheduling Problem (SDVSP). The multiperiod vehicle scheduling problem (MPVSP) within a transportation system where a fleet of homogeneous vehicles delivers products from a central depot to retailers was introduced by Kim and Kim (1999). The objective of the MPVSP is to minimize transportation costs for product delivery and inventory maintenance costs during the planning horizon. Recently, motivated by the problem faced by a company that provides installation and maintenance services for electrical infrastructures, Guastaroba et al. (2021) introduced the multiperiod workforce scheduling and routing problem (MP-WSRP). The MP-WSRP requires determining an optimal dispatch plan to meet the given set of jobs by routing several pieces of equipment over a finite planning horizon.

A variant of the SDVSP by considering the problem of the location of multiple cross-docking centers (CDCs) and the scheduling of vehicle routes was studied by Mousavi et al. (2014). This work developed two mixed-integer linear programming (MILP) models integrating CDC location decisions and vehicle route schedules. Similarly, Moghadam et al. (2014) considered a problem of the routing and scheduling of vehicles in a network composed of suppliers, customers, and cross-docking. In this work, a nonlinear mathematical model of mixed-integer programming was proposed. Dondo and Cerdá (2015) consider the cross-docking vehicle routing problem (CDVRP). This paper presents a mathematical formulation of MILP for the CDVRP to determine the routing and scheduling of a fleet of hybrid vehicles, the assignment of the dock door, the docking sequence of trucks, and the travel time required to move the products. Recently, Correa et al. (2020) considered the problem of scheduling trucks in cross-docking through the design of a MILP model. In particular, they seek to determine a schedule of entry and exit of trucks in a dock area to minimize the time from when the first inbound truck arrives until the last outbound truck leaves (makespan).

Multiobjective methods for vehicle routing and scheduling problems within supply chain problems have been studied recently by Rahbari et al. (2019), Goodarzi et al. (2020), and Shahabi-Shahmiri et al. (2021). A bi-objective MILP model for the vehicle routing and scheduling problem with cross-docking for perishable products has been studied by Rahbari et al. (2019). In this work, two robust models are developed when the travel time of the outbound vehicles and the freshness-life of the products is uncertain. Goodarzi et al. (2020) address a vehicle routing problem with cross-docking (VRPCD) by considering truck scheduling and splitting pickup and delivery orders with time windows at supplier and retailer locations. Two conflict objectives are considered: minimization of the total operational cost and the sum of the maximum earliness and tardiness. Finally, a new multiobjective mixed-integer programming (MIP) model is proposed for scheduling and routing heterogeneous vehicles carrying perishable goods across multiple cross-docking systems. The main objectives of the developed model are to reduce distribution costs, accelerate distribution processing time, and maximize the cross-docking network's capacity utilization rate. A review of the state of the art on similar problems related to scheduling in maritime routing can be found in Christiansen et al. (2004).

Selection and assigning orders for vehicle scheduling problems within supply chain problems were considered by García and Lozano (2005), Chen and Pundoor (2006), and Liu et al. (2018). García and Lozano (2005) develop a problem of the selection and planning of orders of a manufacturing plant for immediate delivery to its customers. The most important constraints for this study are the number of vehicles available and the time windows that must be delivered. Chen and Pundoor (2006) considered an order allocation and planning problem within a supply chain. The authors study the allocation and planning of the orders from a static deterministic approach and look for a schedule to deliver the complete orders to each DC after production. Liu et al. (2018) addressed the problem of assigning orders for delivery in the last mile. Historical delivery data are studied to improve the "on-time" performance in fast food delivery services.

Recently published works dealing with vehicle and scheduling problems were considered by Melchiori et al. (2022), which considered the problem of raw material allocation, routing, and scheduling. The authors proposed a model that involves an arc-based formulation for routing and a time grid discretization, including the definition of loading and unloading shifts for scheduling. Similarly, Cota et al. (2022) analyzed the integrated problem in which trucks' scheduling in a cross-docking center with multiple docks is combined with the associated open vehicle routing problem, called Open Vehicle Routing Problem With Cross-Docking (OVRPCD). A mathematical model and two heuristics approach to solve the OVRPCD were proposed.

On the other hand, data processing considers collecting and manipulating data elements to produce meaningful information. This information could be transformed into knowledge and, subsequently, a decision (Han et al. 2011). Data processing can be divided into five stages: collection, preparation, processing, analysis, and storage (French 1996). Some of the stages of data preparation include cleaning the DBs, transforming the variables or attributes, extracting attributes, and selecting attributes (Han et al. 2011). The problem of null values represents the loss of possibly relevant information, hindering the data analysis. Some techniques to treat this are eliminating attributes, tuples, and the imputation of data (Han et al. 2011). Another phase of DB cleaning is the treatment of out-of-range values. Out-of-range values are data that are far from the rest, and their presence may be due to measurement errors or a data distribution with high kurtosis. Some techniques for identifying these values include using dispersion measures, such as the mean and standard deviation, and the quartile method (Han et al. 2011). One tool for processing information is data mining using clustering or grouping. There are various clustering techniques and algorithms, such as the K-means technique. This method groups data into n groups with equal variance, in which each observation belongs to the group with the closest mean. This result can be achieved using Lloyd's algorithm or Elkan's algorithm (Pedregosa et al. 2011). Likewise, a method for the validation of clusters is the elbow method. The elbow method is a heuristic used to determine the optimal number of clusters and generally consists of graphing the explained variation as a function of the clusters and choosing the elbow of the curve as the number of groups to be used (Syakur et al. 2018).

We have yet to find a similar combining optimization and data mining approach to deal with a scheduling vehicle problem from the literature review. Besides, the published papers generally use heuristic or metaheuristic approaches for large-size real instances, including real instances. Finally, only some studies have studied an efficient mathematical formulation, including several real constraints.

### Contributions

We introduce a new problem belonging to the class of routing and scheduling problems within a finite period. The new problem is the Rich Single-Depot Vehicle Scheduling and Routing Problem (RSD-VSRP), a variant of the well-known Single Depot Vehicle Scheduling Problem (SDVSP). Different realistic constraints of real complex problems have been considered. The main novel characteristics of our problem are the multiperiod environment and the dispatch of vehicles, including fundamental considerations for an entire operation of a DC. The solution scheme is flexible and could be adapted for any company with a delivery process from DCs. Also, the proposed MILP mathematical model is innovative and efficient in solving large-size problems. The former approach can solve the RSD-VSRP using a two-phase scheme, combining data mining and optimization strategies. Unlike previous similar studies, we propose a scheme to find the optimal solution for real large instances. Finally, extensive computational experiments are performed to test the efficiency of the proposed methodology with a case study based on data provided by a Chilean company.

## Proposed methodology

This paper seeks to optimize routing and scheduling for a company that operates a two-echelon distribution system, delivering a set of products from a DC to a set of independent (penultimate) customers who, in turn, deliver the "last mile" to independent, ultimate customers. Routing involves assigning an ordered subset of customers to each delivery truck, and scheduling involves setting delivery times to observe time windows set by customers. The solution methodology is composed of two phases. In the first phase, data processing must be performed to extract the parameters and sets of the proposed model. The second phase corresponds to the design of the destination nodes' allocation model to determine the product distribution schedule.

### Problem description

We have considered a Rich Single-Depot Vehicle Scheduling and Routing Problem (RSD-VSRP), a variant of the well-known Single Depot Vehicle Scheduling Problem (SDVSP), considering rich constraints. The SDVSP could be modeled by a complete graph $G=(V,E)$ with vertices$V={N}_{1}\cup {N}_{2}\cup \left\{s,t\right\}$. Let be ${N}_{1}= \{{i}_{e}, \forall i \in N\}$ and${N}_{2}= \{{i}_{s}, \forall i \in N\}$, where each trip $i$ is represented by two nodes: a starting node${i}_{s}$, and ending node${i}_{e}$, respectively. Besides, the depot is represented by nodes $s$ (depot as starting node) and $t$ (depot as ending node), with $N = \{1, 2, . . . , n\}$ as the set of trips, numbered according to increasing starting time. Note that trips $i$ and $j$ constitute a compatible pair of trips if the same vehicle reaches the starting point of trip $j$ after it finishes trip$i$.

The set of edges $E =(s \times {N}_{2})\cup Z\cup D\cup ({N}_{1}\times t)\cup (s,t)$ considers the subset $Z=\left\{({i}_{e},{j}_{s})\left|ct\left(i,j\right)=1, i\in N,j\in N\right.\right\}$, where $ct\left(i,j\right)$ is a binary parameter characterizing a compatible pair of trips. If $ct\left(i,j\right)=1$, trips $i$ and $j$ confirm a compatible pair of trips, 0 otherwise. Besides, let $D=\left\{({i}_{s},{i}_{e})\left|i\in N\right.\right\}$ be the set of edges connecting the starting to the ending of a trip. Note that the upper bounds of edges in $D$ are 0, and the edge flow is 0. A path from $s$ to $t$ in $G$ represents a feasible vehicle schedule.

The associated costs ($${r}_{ij})$$ to an edge $$(i,j)\in E$$ are defined as follows:

$$r_{ij} = \left\{ \begin{gathered} {\text{travel}}\;{\text{time}}\;{\text{of}}\;{\text{the}}\;{\text{edge}} + {\text{the}}\;{\text{waiting}}\;{\text{time}}\;{\text{till}}\;{\text{the}}\;{\text{starting}}\;{\text{of}}\;{\text{future}}\;{\text{trip}}\;j\quad \forall \left( {i,j} \right) \in Z \hfill \\ 0\quad \quad \quad \quad \quad \quad \quad \quad \quad \quad \quad \quad \quad \quad \quad \quad \quad \quad \quad \quad \quad \quad \quad \quad \quad \quad \quad \quad \;\forall \left( {i,j} \right) \in D \hfill \\ {\text{travel}}\;{\text{time}}\;{\text{of}}\;{\text{the}}\;{\text{edge}} + {\text{fixed}}\;{\text{vehicle}}\;{\text{cost}}\quad \quad \quad \quad \quad \quad \quad \quad \quad \quad \quad \quad \forall \left( {s \times N_{2} } \right) {\text{or}} \left( {N_{1} \times t} \right) \hfill \\ 0\quad \quad \quad \quad \quad \quad \quad \quad \quad \quad \quad \quad \quad \quad \quad \quad \quad \quad \quad \quad \quad \quad \quad \quad \quad \quad \quad \quad \;\forall \left( {i,j} \right) \in \left( {s,t} \right) \hfill \\ \end{gathered} \right.$$

A number ${B}_{i}$ is associated with each node $i \in V$ indicating supply or demand, depending on whether ${B}_{i}>0$ or ${B}_{i}<0$, as follows:

- A demand node with ${B}_{i}=-1$ is set for each node $i \in {N}_{2}$;
- A supply node with ${B}_{i}=1$ is set for each node $i \in {N}_{1}$;
- A supply node with ${B}_{s}=\left|N\right|$ is set for the starting depot $s$;
- A demand node with ${B}_{t}=-\left|N\right|$ is set for the ending depot $t$.

Since the demand of the starting node of a trip (${i}_{s}$) is one and the upper bound of its only outgoing edge is 0, each trip can only receive a unitary flow. The supply at the end of a trip (${i}_{e}$) is 1, and each trip can send at most one-unit flow. Note that each trip must be covered only once, and there are $n$ trips to be fulfilled. Thus, $n$-units of flow are sufficient to serve them, and the supply of the starting depot is set to $n$. If the number of vehicles required to serve the collection trips is less than $n$, the remaining supply of the starting depot $s$ is sent to the ending depot $t$ via the edge $(s,t)$, which does not generate cost since the cost of the arc $(s,t)$ is 0.

The mathematical model of the SDVSP could be formulated as a minimum-cost flow problem. The decision variable, ${y}_{ij}=1 \forall (i,j)\in E$ if the edge trip $j$ is performed once the trip $i$ is developed. The mathematical programming formulation of the SDVP based on flow is defined as follows:

1 $$\mathrm{min}\sum_{\left(i,j\right) \in E}{r}_{ij}{y}_{ij}$$

Subject to

2 $$\sum_{j: \left(i,j\right) \in E}{y}_{ij}-\sum_{j: \left(j,i\right) \in E}{y}_{ij}=1\;\forall i\in {N}_{1}$$

3 $$\sum_{j: \left(i,j\right) \in E}{y}_{ij}-\sum_{j: \left(j,i\right) \in E}{y}_{ij}=-1\;\forall i\in {N}_{2}$$

4 $$\sum_{j: \left(i,j\right) \in E}{y}_{ij}-\sum_{j: \left(j,i\right) \in E}{y}_{ij}=\left|N\right|\;\forall i=s$$

5 $$\sum_{j: \left(i,j\right) \in E}{y}_{ij}-\sum_{j: \left(j,i\right) \in E}{y}_{ij}=-\left|N\right|\;\forall i=t$$

6 $$0\le {y}_{ij}\le 1\;\forall \left(i,j\right)\in E\backslash (s,t)\backslash D$$

7 $${y}_{st}\ge 0$$

8 $${y}_{ij}=0\;\forall \left(i,j\right)\in D$$

The objective function (1) minimizes the total operating and fixed vehicle costs. Constraints (2, 3, 6, and 8) ensure that each trip is served only once. Constraints (4, 5, and 7) guarantee the sufficiency of vehicles. Unfortunately, this formulation cannot be directly applied to the considered problem due to different objective function and additional constraints that must be considered:

(i) Minimization of the number of trips;
(ii) Each vehicle could perform only one daily route;
(iii) Special frequencies for scheduling deliveries are considered;
(iv) Working hours cannot be exceeded, and;
(v) Penalty aspects per service time per supermarket visited on the day.

Consequently, the RSD-VSRP should be treated as a variant of the classical SDVSP. We found works with different realistic constraints in the literature review. However, real constraints generally increase the difficulty of solving an RSD-VSRP for large-size instances. Therefore, using heuristic or metaheuristic approaches is the standard approach to dealing with a real problem. We have solved the problem optimally for a real instance. Also, combining data mining and optimization schemes to solve large real instances optimally for an RSD-VSRP has yet to be proposed. Therefore, we introduced the problem and proposed a two-stage scheme to solve it. The stages are explained as follows:

### Phase 1. Information processing

The collection, processing, and refinement of data and information is key to obtaining representative and quality inputs for the optimization model (Phase 2). The problem, when addressed with a static approach, is developed with historical data from any company. However, as seen later, some parameters are obtained through information surveys using qualitative methods. The assumptions and scope in Phase 1 are as follows:

- We work with specific data for a specific and fixed period, so the results represent that single period. This fact does not imply that the behavior of the data can change in other periods.
- The data analyzed correspond to the pandemic period of COVID-19, which may be different from previous periods given the health contingencies caused by the SARS-CoV-2 virus.
- The data and information related to the service times and schedules of the destination nodes are obtained by consulting the perceptions and experiences of the operating personnel of the DC.

Next, the processes performed to define the parameters and main sets for the allocation model are described. The problem is that a distribution company of a varied range of products must make deliveries to commercial nodes with different characteristics and requirements. Given the stochastic behavior of market demands and contextual factors, the nodes to which a company dispatches vehicle can maintain consistency and volume or vary over time. In this way, an annual historical period should be selected since it is sufficient to collect valid data on the current destination nodes, their demand, and dispatch frequency.

The first stage of data processing considers the preparation and cleaning of the DBs to obtain the parameters of the destination nodes, frequency of visits, and demand. In the DB analysis, duplicate and conflicting information can be obtained, causing the number of kilograms delivered and trips made to increase. Thus, the attributes that do not provide information to the case are eliminated. Finally, the atypical nodes are treated, where the data that present several kilograms dispatched or several performed trips far from the concentration of the data are eliminated using the quartiles method.

#### Selection of destination nodes

The selection of the destination nodes is the basis of the problem for Phase 2. Therefore, it is necessary to select the most relevant nodes for the model. According to the selected characteristics, the relevant destination nodes have a monthly demand greater than $\partial$ or those that are receive at least $\vartheta$ visits in a month. Following the guidelines of this constraint, two conditions are proposed (Algorithms 1 and 2) for the extraction of the destination nodes.

Algorithm 1 First condition for the selection of destination nodes.
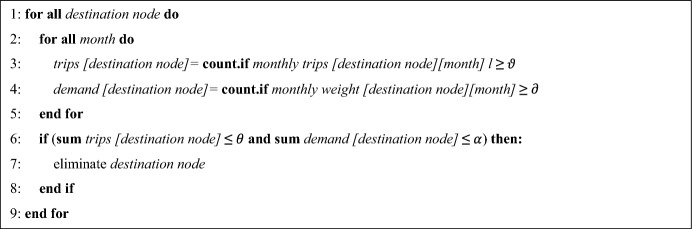


Source: Owner.

Algorithm 2 Second condition for the selection of destination nodes.
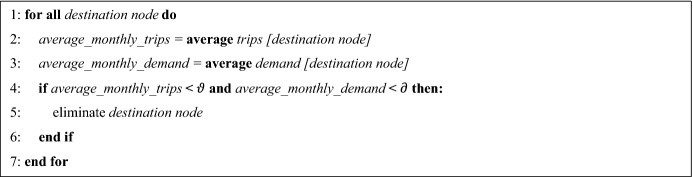


Source: Owner.

The conditions described have the objective of maintaining destination nodes that present consistent demand for products or number of visits and, in turn, comply with the constraint in a general way, requiring that the monthly averages of the demand or trips are at least $\partial$ [kilograms/month] or $\vartheta$ [trips/month], respectively (Condition 2).

#### Frequency of visits

Clustering techniques are used to extract the frequency of visits to the destination nodes. In this way, the distance between the monthly number of visits per destination node, the absolute frequency, and the frequency established for the allocation model is reduced. Therefore, the two questions of this phase are as follows: how many clusters for trip frequency should be established? Moreover, what should the trip frequencies be? The algorithm used to create the clusters is K-means. The optimal number of clusters must be calculated using the elbow method with the K-means algorithm in the first instance. The centroids of the clusters represent the visitation frequencies of the destination nodes.

#### Other parameters

The demand is calculated as the monthly average of kilograms of products delivered to the selected destination nodes. Thus, trip demand for each node is obtained from the quotient between the monthly demand and the frequency of the monthly visit. The schedules of the destination nodes (time windows) are determined through the experience of the company. Two groups of nodes were created according to their daily hourly availability, where one group represents the "supermarket" type destination nodes, and the other represents all nodes of another type.

The georeferencing of the nodes can be obtained through the "Geocoding" API of Google Maps Platform®. A Python code is generated to communicate with the API and obtain the nodes' coordinates using their respective addresses, which are obtained from the DB. Before this process, typing errors are reviewed and corrected to avoid erroneous coordinates. The 225 nodes are scattered in three provinces, Arauco, Concepción, Itata, and 20 communes (similar to municipalities).

The intracommunal time represents an estimate of the time it takes to travel between destination nodes in a given commune. This value is the same for all trips between nodes in a commune. A Python code is generated to communicate with the "Distance Matrix" API of Google Maps Platform®, giving it the coordinates of the destination nodes of a commune and repeating this for each commune. The times requested from the API are unidirectional, assuming that the difference in the route between one destination node and another, in both directions, is approximately the same. In this way, the number of travel times per commune delivered by the program is type (n − 1)! where n is the number of destination nodes per commune. Finally, the average travel time per commune is calculated.

The intercommunal time is the estimated travel time between communes in a given province. This value is the same for all the communes of the same province. The first stage consists of calculating the centroids of the communes according to the locations of the nodes. Thus, the times are obtained from the concentrations of the nodes and not from the centers of the communes. Subsequently, for intracommunal times, a Python code is created to communicate with the "Distance Matrix" API, giving it the coordinates of the centroids of the communes; this is repeated for each of the provinces. As in the previous process, the times requested from the API are unidirectional, assuming that the routes between communes in both directions are approximately the same. For the Province of Concepción, the travel times between commune centroids greater than 40 min are eliminated, preventing the parameter from being influenced by trips that are not made in reality, as they are excessive distances. Finally, the average travel time between commune centroids is calculated for each province.

The service time is estimated using a two-stage qualitative tool. In the absence of historical information on the times that drivers and delivery assistants take to deliver products, drivers with more experience in the company are interviewed to determine their general perceptions about this factor. Through the carriers' responses, a questionnaire is designed to collect more specific information about their experiences and convert these into figures.

### Phase 2. Optimization model for the dispatch schedule

Phase 2 of the proposed methodology corresponds to the design of a mixed-integer linear optimization model for the distribution schedule of the DC. This model is built mainly using allocation and scheduling rules that determine the customer assigned to a vehicle on a given day of the finite planning horizon. The parameters and sets of the model are obtained in Phase 1 of the methodology, to which constants and constraints are added based on the work context of the problem considered, such as working hours, fleet size, and vehicle capacity. The model is constructed for a monthly planning horizon, where four weeks are considered, including business days from Monday to Friday.

The proposed mathematical model considers the following assumptions and scopes:

- At the destination nodes, it is assumed that transportation times are constant for all vehicles and are not influenced by external agents.
- Any logistics operation before the transport of products does not influence the scheduling.
- A homogeneous vehicle fleet is considered.
- It is assumed that all orders are fully delivered. Indeed, there are no products that must be returned to the DC or re-dispatched.
- The transportation time from the DC to the first visited node by a vehicle is neglected, and from the last visited destination node by this vehicle returning to the DC.
- Only the weight of products is considered, without considering product volume and the number of pallets the vehicle could carry.
- The time windows for each vehicle are calculated from the service time and the penalty time of the destination node without considering the travel times.

The proposed model's sets, parameters and indexes, decision variables, objective function, and constraints are below.

#### Sets, parameters and indexes

Tables 1, 2 and 3 show the related information for the proposed model.

**Table 1:** Indexes for the proposed model. Source: Owner

$i$	Destination nodes, $i\in N$
$j$	Days, $j\in D$
$k$	Vehicles, $k\in K$
$c$	Communes,$c\in C$
$p$	Provinces, $p\in P$

**Table 2:** Destination nodes mapping sets. Source: Owner

$N=\left\{{n}_{1},{n}_{2},\dots ,{n}_{i}\right\}$	Set of destination nodes $i$
$D=\left\{{d}_{1},{d}_{2},\dots ,{d}_{j}\right\}$	Set of days $j$
$K=\left\{{veh}_{1},{veh}_{2},\dots ,{veh}_{k}\right\}$	Set of vehicles $k$
$A=\left\{{dem}_{1},{dem}_{2},\dots ,{dem}_{i}\right\}$	Set of demand for each destination node $i$
$C=\left\{{com}_{1},{com}_{2},\dots ,{com}_{c}\right\}$	Set of communes $c$
$P=\left\{{prov}_{1},{prov}_{2},\dots ,{prov}_{p}\right\}$	Set of provinces $p$
$S=\left\{{sup}_{1},{sup}_{2},\dots ,{sup}_{i}\right\} S \subseteq N$	Set of nodes $i$ denominated supermarkets
$T=\left\{{ts}_{1},{ts}_{2},\dots ,{ts}_{i}\right\}$	Set of service time for each node $i$
$ESPI=\left\{10, 30, 56, 116, 140\right\},$	Set of special nodes with high frequency
$ESPJ=\{1, 3, 5, 6, 8, 10, 11, 13, 15, 16, 18, 20$}	Set of days for which the special nodes must be visited

**Table 3:** Parameters for the proposed model. Source: Owner

$$f\left(i\right)= \left\{\begin{array}{c}10, if\;the\;visit\;frequency\;is\;every\;two\;days\\ 5, if\;the\;visit\;frequency\;is\;every\;four\;days\\ 2, if\;visit\;frequency\;is\;biweekly\end{array}\right.$$	Visit frequency function for each destination node $i.$
$$g\left(j,f\left(i\right)\right)= \left\{\begin{array}{c}\widehat{j} mod2, f\left(i\right)=10\\ \widehat{j} mod4, f\left(i\right)=5\\ \widehat{j} mod10, f\left(i\right)=2\end{array}\right.$$	Function that replicates the visits to node $i$ according to it frequency $f\left(i\right)$
$${com}_{ic}=\left\{\begin{array}{c}1,if\;the\;node\;i\;belongs\;to\;the\;commune\;c\\ 0,\;otherwise\end{array}\right.$$	Matrix that indicates the destination node $i$ belonging to the commune $c$
$${prov}_{cp}=\left\{\begin{array}{c}1,if\;the\;commune\;c\;belongs\;to\;the\;province\;p\\ 0,otherwise\end{array}\right.$$	Matrix that indicates the commune $c$ belonging to the province $p$
${dem}_{i}$	Demand for each destination node $i$
${ts}_{i}$	Service time for each destination node $i$
${t\_intra}_{c}$	Estimated time of route between each destination node $i$ belonging to the commune $c$. This time is calculated from the mean of distances between the destination nodes of a commune $c$
${t\_inter}_{p}$	Estimated time of route between each commune $c$ belonging to the province $p$. This time is calculated from the mean of distances between centroids of the communes belonging to the province $p$. The centroids are calculated based on the nodes of each commune $c$
$Cap$	Capacity for each vehicle
$T\_sp$	Extra service time if more than one supermarket is assigned to a vehicle on a route
$WH$	Maximum number of daily working hours for each vehicle
$j\_morn$	Maximum number of working hours in the morning shift for each vehicle
$j\_afte$	Maximum number of working hours in the afternoon shift for each vehicle
$bigM$	It is a significant number, equivalent to the sum of the demand values of all destination nodes

#### Decision variables

Table 4 shows the decision variables for the proposed model.

**Table 4:** Decision variables for the proposed model. Source: Owner

$${X}_{ijk}=\left\{\begin{array}{c}1\\ 0\end{array}\right.$$	, if the destination node $i$ is visited the day $j$ by the vehicle $k$ , Otherwise
$$M{X}_{ijk}=\left\{\begin{array}{c}1\\ 0\end{array}\right.$$	, if the destination node $i$ is visited the day $j$ by the vehicle $k$ in the morning shift , otherwise
$$T{X}_{ijk}=\left\{\begin{array}{c}1\\ 0\end{array}\right.$$	, if the destination node $i$ is visited the day $j$ by the vehicle $k$ in the afternoon shift , otherwise
$${Y}_{cjk}=\left\{\begin{array}{c}1\\ 0\end{array}\right.$$	, if the commune $c$ is visited the day $j$ by the vehicle $k$ , otherwise
$${Q}_{pjk}=\left\{\begin{array}{c}1\\ 0\end{array}\right.$$	, if the province $p$ is visited the day $j$ by the vehicle $k$ , otherwise
${Z}_{cjk}$	Number of trips between nodes of commune $c$ visited the day $j$ by the vehicle $k$
${W}_{pjk}$	Number of trips between nodes of province $p$ visited the day $j$ by the vehicle $k$
${V}_{pjk}$	Number of communes of province $p$ visited the day $j$ by the vehicle $k$
${SP}_{jk}$	Number of supermarkets visited the day $j$ by the vehicle $k$

#### Mathematical formulation

Objective function

9 $$Minimize\;Trips =\sum_{p\in P}{V}_{pjk}$$

Constraints

10 $$\sum_{k\in K}\sum_{j\in D}{X}_{ijk}=f\left(i\right)\;\forall i \in N$$

11 $$\mathop \sum \limits_{k \in K} X_{ijk} = \mathop \sum \limits_{k \in K} X_{{i\hat{j}k}}\;\forall i \in N,\hat{j} \in g\left( {j,f\left( i \right)} \right),j \in D$$

12 $$\sum_{k\in K}{X}_{ijk}\le 1\;\forall i\in N,j\in D$$

13 $$\sum_{k\in K}{X}_{ijk}=1\;\forall i\in ESPI,j\in ESPJ$$

14 $$\sum_{k\in K}{X}_{ijk}=0\;\forall i\in ESPI, j \in D-\{ESPJ\}$$

15 $$\sum_{i\in N}t{s}_{i}{X}_{ijk}+\sum_{c\in C}{{{t}_{intra}}_{c}Z}_{cjk}+\sum_{p\in P}{{t}_{inter}}_{p}{W}_{pjk}+{T\_spSP}_{jk}\le WH\;\forall j\in D,k\in K$$

16 $$\sum_{i\in N}{{com}_{ic}X}_{ijk}\le bigM {Y}_{cjk}\;\forall c\in C,j\in D,k\in K$$

17 $$\sum_{c\in C}{prov}_{cp}{y}_{cjk}\le bigM {Q}_{pjk}\;\forall p\in P,j\in D,k\in K$$

18 $$\sum_{p\in P}{Q}_{pjk}\le 1\;\forall j\in D, k\in K$$

19 $${Z}_{cjk}\ge \sum_{i\in N}{({com}_{ic}X}_{ijk})-1\;\forall j\in D,k\in K,c\in C$$

20 $${W}_{pjk}\ge \sum_{c\in C}({prov}_{cp}{y}_{cjk})-1\;\forall j\in D,k\in K,p\in P$$

21 $${V}_{pjk}\ge \sum_{c\in C}({prov}_{cp}{y}_{cjk})\;\forall j\in D,k\in K,p\in P$$

22 $${SUP}_{jk}\ge \sum_{i\in S}({X}_{ijk})-1\;\forall j\in D,k\in K$$

23 $$\sum_{i\in N}{{ dem }_{i}X}_{ijk}\le Cap\;\forall j\in D,k\in K$$

24 $$\sum_{i\in S}({ts}_{i}{X}_{ijk})+\sum_{i\in N-\{S\}}{({ts}_{i}MX}_{ijk})+{T\_spSUP}_{jk}\le {j}_{morn}\;\forall j\in D,k\in K$$

25 $$\sum_{i\in N-\{S\}}{({ts}_{i}TX}_{ijk})\le {j}_{afte}\;\forall j\in D,k\in K$$

26 $${MX}_{ijk}+{TX}_{ijk}= {X}_{ijk}\;\forall i\in N, j\in D,k\in K$$

27 $${X}_{ijk}, {Y}_{cjk}, {Q}_{pjk},T{X}_{ijk},M{X}_{ijk}\in \left\{\mathrm{0,1}\right\}\;\forall i\in N,j\in D, k\in K, c\in C,p\in P$$

28 $${SUP}_{jk},{Z}_{cjk}, {W}_{pjk},{V}_{pjk}\in {\mathbb{N}}\forall j\in D, k\in K, c\in C,p\in P$$

Equation (9) shows the objective function of the mathematical model, which consists of minimizing the total number of trips to communes that vehicles make in the monthly period. This Equation indirectly indicates the number of intercommunal trips made in the month. Equation (10) determines the frequency of participation, ensuring that the number of visits in the month associated with each destination node is met. Constraint (11) establishes the periodicity, indicating that the destination node $i$ visited on day $j$ must receive the next delivery on day $\widehat{j}$, where $\widehat{j}$ is given by $g\left(j,f\left(i\right)\right)$. This function ensures that the required periodicity of visits to the destination node is met. Equation (12) establishes that vehicle k can make a maximum of one daily route.

Equations (13) and (14) define the special frequencies for scheduling deliveries. In particular, Eq. (13) predefines the delivery days of particular destination nodes with a high frequency of visits. These days are Monday, Wednesday, and Friday of every week of the month. Equation (14) predefines the days that do not include distribution to the special destination nodes with a high frequency of visits. These days are Tuesday and Thursday of every week of the month. Constraint (15) establishes that working hours cannot be exceeded. These are defined from the sum between the service time of the destination node ${ts}_{i}$, the estimated route times between each destination point $({t\_intracommunal}_{c}$), the estimated route times between each commune of the destination nodes (${t\_intercommunal}_{p}$), and the additional time that is activated when more than one supermarket is visited ($T\_supermarket$). The travel time from the DC to the first destination point is not considered, nor is the travel time back to the DC.

Equation (16) establishes that if a destination node of commune $c$ is visited, then commune $c$ is also visited. Constraint (17) determines belonging to the province, establishing that if a commune of province $p$ is visited, then province $p$ is also visited. Equation (18) establishes that a province can be visited at most once by a vehicle $k$ on day $j$.

The number of trips between nodes and communes is constrained by (19) and (20), respectively. Equation (19) establishes the number of trips between nodes of commune $c$ that are visited on day $j$ with vehicle $k$ (intracommunal travel). This situation can also be referred to as the number of arcs between destination nodes generated by vehicle $k$ on day $j$ in commune $c$. Constraint (20) establishes the number of trips between the communes in province $p$ that are visited on day $j$ with vehicle $k$ (intercommunal trips). This situation can also be referred to as the number of arcs between communes generated by vehicle $k$ on day $j$ in province $p$.

The numbers of communes and supermarkets visited are constrained by (21) and (22). Equation (21) establishes the total number of communes in province $p$ visited on day $j$ with vehicle $k$. Constraint (22) establishes the additional service time penalties per supermarket visited on day $j$ with the vehicle $k$. Equation (23) establishes that the capacity of the vehicle cannot be exceeded. It is defined as the sum of the demands of destination nodes $i$ that are visited on day $j$ by the vehicle $k$. The time windows are restricted by Eqs. (24–26). In particular, the vehicle can visit a destination node only within the reception hours. For this, the time that the vehicle occupies in the morning shift and afternoon shift is estimated. The time is calculated from the service times of the destination nodes for the morning shift plus the additional penalty of the constraint “number of supermarkets visited” and for the afternoon shift only from the service times of the destination nodes. Finally, Constraints (27) and (28) determine the nature of the variables.

## Design of experiments and computational results

The model of the second phase is programmed using the JuMP mathematical optimization language incorporated in the programming language Julia v1.6.2 and is solved using the Gurobi v9.1 *solver.* In addition, the *XLSX.jl* and *DataFrames.jl* packages were used for data management. A computation time limit of 7200 s is established for the case study. The experiments are developed in Red Hat Enterprise Linux 8® with an Intel® Core™ i5-3570 CPU @ 3.40 GHz and 16 GB of RAM.

### Company case study

**Fig. 1:**
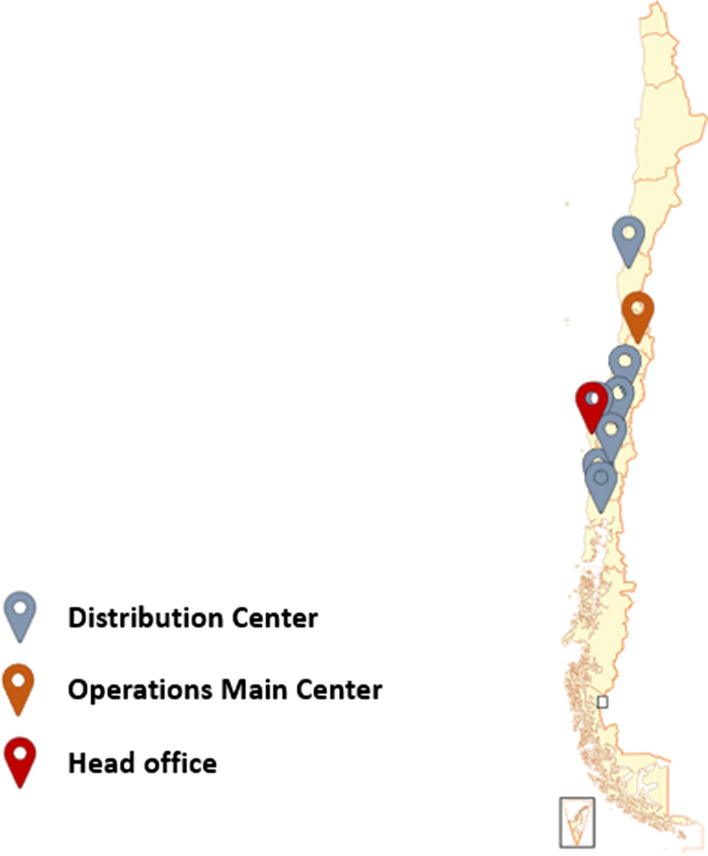
Company facilities. Source: Own elaboration based on the case of study

The case study company is a family business of road freight transport and distribution logistics of nonperishable products founded in 2001. Its DCs are strategically distributed throughout Chile. The operations center is located in the city of Santiago. Finally, the headquarters are located in Concepción. Currently, the DCs are located in Osorno, Valdivia, Temuco, Concepción, Chillán, Talca and La Serena (Fig. 1).

The customer's portfolio is currently composed of approximately 220 customers. The company delivers products to supermarkets, distributors, retailers, wholesalers, among others. The route calendar is preset, where the frequency of visits is defined according to the commune of the destination point. The strategic process of route creation is planned annually and is carried out based on a fundamental estimation of the demands of the destination nodes and, consequently, the demands of the communes. Once the orders are loaded, the vehicle goes to the last participant in the supply chain to dispatch the vehicles. The assignment of the orders to the vehicles is made by the head of the agency, while the vehicles' route is defined by the drivers based on their experience.

A relevant factor for this process is the reception schedule of customer loads. National supermarket chains have strict reception hours, between 8:00 and 13:00, while the other destination nodes usually have extended reception hours, between 8:00 and 18:00, with closing times generally between 13:00 and 15:00.

The company has a wholly outsourced delivery fleet, which reduces the fixed costs in the operations area. There are seven transportation services, which translates into a fleet size of 10 vehicles. The vehicles have an average capacity of 5000 kg and eight pallets in total, with an average volume of approximately 31.25 m^3^.

### Results of phase 1

This section shows the main results of the data collection and processing phase. Initially, the DB contains 29,496 invoices. The first attribute transformation results in 1374 destination nodes. This new DB is called the “Destination nodes” DB and has the following attributes:

- (1): Unique keyword for each destination node from *Codpg*
- (2): Delivered monthly weight for each destination node
- (3): Monthly trips to each destination node

Tables 5 and 6 show the initial situation and the final situation after cleaning the destination nodes of DB.

**Table 5:** Results cleanup destination nodes DB. Source: Owner

	Current Situation	Deleted duplicate values	Out of range values
Deleted nodes	–	97	10
Total nodes	1374	1277	1267

**Table 6:** Destination nodes selection results. Source: Owner

	Current Situation	First Condition	Second Condition
Deleted nodes	–	1034	8
Total nodes	1267	233	225

**Fig. 2:**
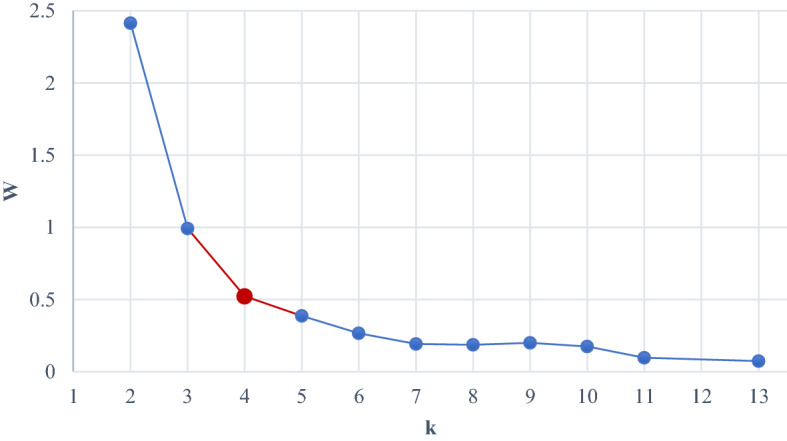
Elbow method for several visit frequencies. Source: Owner

Figure 2 shows the result of the elbow method technique applied to the visit frequencies of the destination nodes, indicating that the optimal number of clusters is four with the K-means method (Syakur et al. 2018). W and k are calculated according to Syakur et al. (2018).

Additionally, the K-Means method is applied to four clusters, providing the frequencies in Table 7, where the number of destination nodes that belong to each group and the total number of visits that must be performed is detailed. These visits are approximated to the nearest integer since the method operates with rational values.

**Table 7:** Monthly frequency visits for destination nodes. Source: Owner

Cluster	Centroid	Frequency (days)	Number of nodes
1	1.9	2	129
2	13.3	13*	5
3	4.9	5	69
4	9.7	10	22
Total visits	883		

*Frequency that approached 12 days for the model to visit the nodes three times a week

The demands of the destination nodes present the characteristics shown in Table 8, where their characteristics, the monthly totals, and the value to be covered are indicated.

**Table 8:** Demands statistic for destination nodes. Source: Owner

Type	Mean [kg]	Minimum [kg]	Maximum [kg]
Demand per visit	685	60	5.000
Monthly demand	2775	200	19.788
Demanda total	624.457		

The demands of the destination nodes present the characteristics shown in Table 8, where their characteristics, the monthly totals, and the value to be covered are indicated.

Table 9 shows the number of destination nodes that are supermarkets and, therefore, belong to time group "1,"; and the destination nodes that do not have an opening hours constraint belong to group "0". The closing time of the destination nodes was not considered to reduce the complexity of the proposed model.

**Table 9:** Time windows for destination nodes. Source: Owner

Schedule ID	Schedule	Number of nodes
0	8:00–18:00	134
1	8:00–13:00	69

**Fig. 3:**
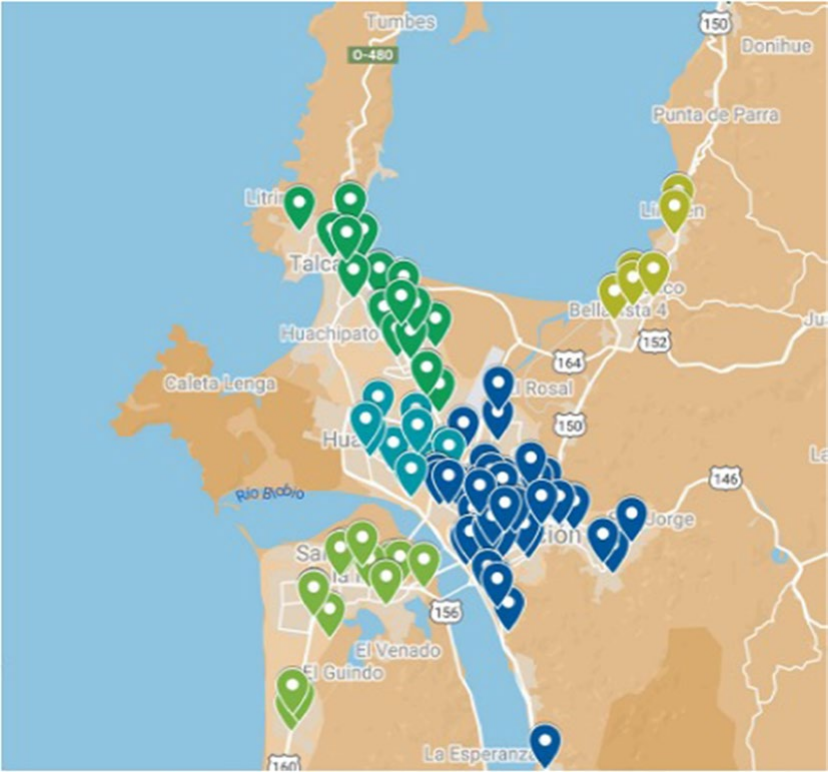
Location of destination nodes. Source: Owner

**Fig. 4:**
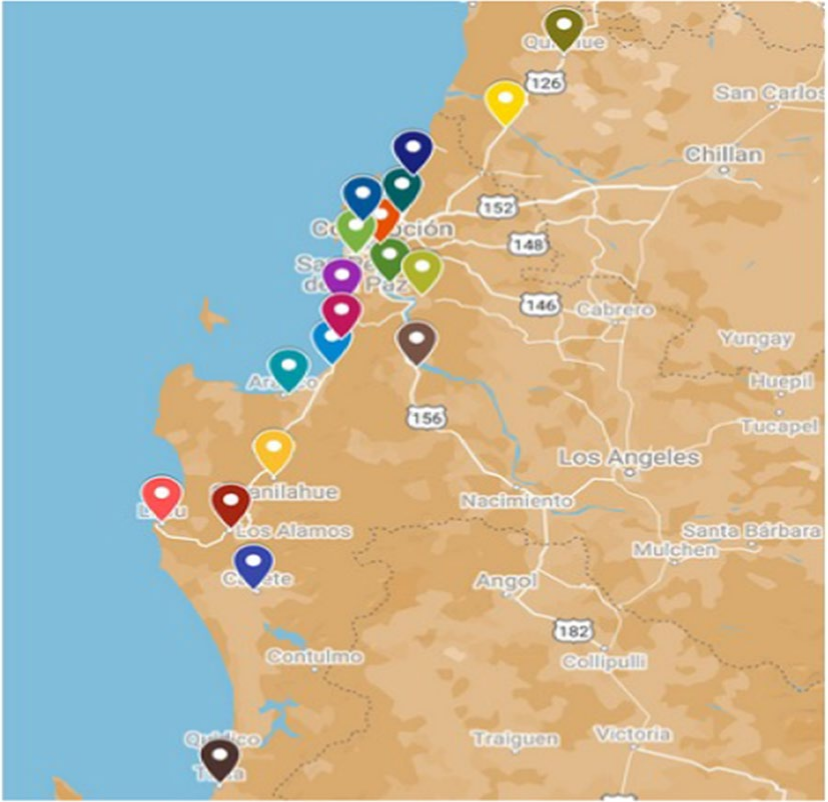
Centroids of commune. Source: Owner

Figure 3 shows the georeferenced destination nodes on the map showing the Concepción Province, Arauco Province, and Itata. Figure 4 shows the calculated centroids of each commune.

Based on the interviews conducted with the carriers and delivery assistants, the service time is segmented into three variables: waiting time before unloading in supermarkets, waiting time before unloading at other types of destination nodes, and unloading time per kilogram. The interviewees emphasized that the waiting times depend on numerous factors, such as the day, the time, the products demand at the commercial nodes, and the order of arrival of the vehicles, among others. For this reason, the estimates do not consider external factors that could affect the service time, except for the arrival time of the vehicles. Concerning the carriers' responses in the questionnaire applied, the importance of visiting the supermarkets first is highlighted given a large number of competing vehicles that must also deliver products; drivers mentioned how the waiting time increases when arriving late. In this way, the waiting time for the supermarket should only be activated if it is not the first visited node; otherwise, the waiting time is insignificant. Additionally, the unloading time involves all the activities of the product delivery process, such as vehicle unloading, document delivery, and product review. The components of the service time are shown in Table 10.

**Table 10:** Service time issue. Source: Owner

Issue	Time
Waiting time for supermarkets**	90 [min]
Waiting time for others customers	0 [min]*
Unloading time per weight	0.025 [min]

*Waiting time at other types of destination nodes was considered negligible

**The waiting time in supermarkets is activated only if more than one supermarket is visited

From the questionnaires, a formula is proposed to calculate the unload time at the destination nodes:

29 $$TD=\frac{TDF}{nc}\times \frac{\sum Invoice size}{nf}$$

where $TDF$: unload time per invoice (minutes). $nc$: number of answered questionnaires. $\sum Invoice size$: sum of weight per delivered invoice. $nf$: number of delivered invoices for the year.

Finally, the formula for the service time considered is the following:

30 $$ts=0.025[\frac{\mathrm{min}}{\mathrm{kg}}]\times trip\_demand[\mathrm{kg}]$$

Table 11 shows the main statistics of the calculated service times.

**Table 11:** Descriptive statistics of service times. Source: Owner

	Mean [min]	Minimum [min]	Maximum [min]
Service time	17	2	38

### Results for phase 2

In Phase 2 of the methodology, service times were linked with the additional waiting time of supermarkets. The results of the proposed model are shown in Table 12.

**Table 12:** Final status of the assignment model for the case of study. Source: Owner

Instance	Size	Trips	Time	Lower bound	Gap
Case of study	N = 225	209	7200 [s]	155	25.84%

**Fig. 5:**
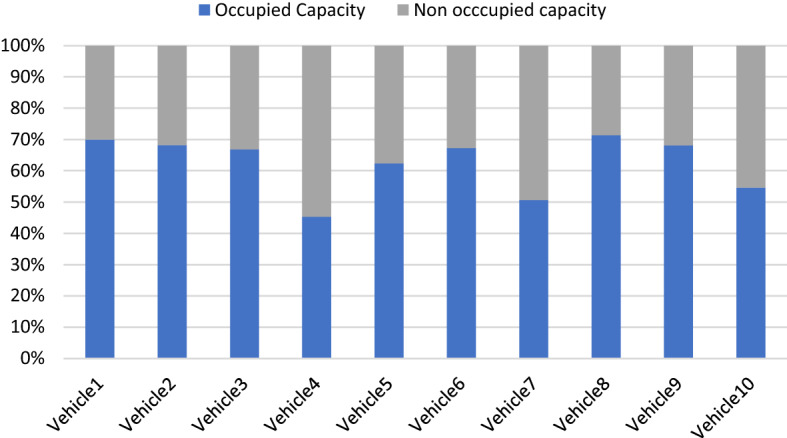
Percentage of occupancy per vehicle. Source: Owner

Figure 5 shows the occupancy percentage of each vehicle obtained in the monthly period.

**Fig. 6:**
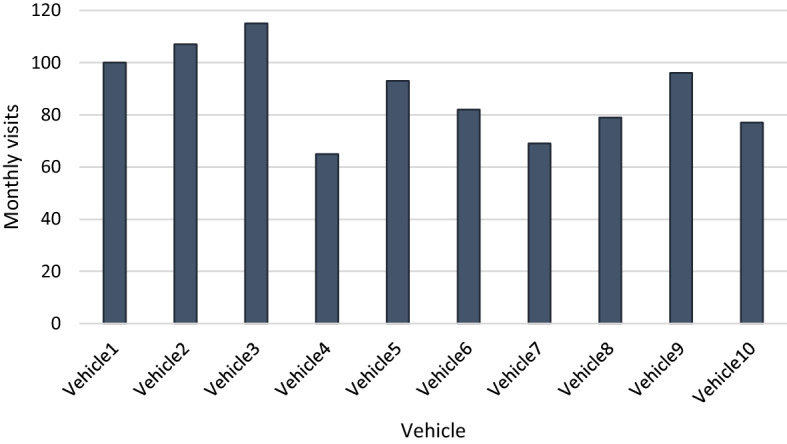
Monthly visits for each vehicle. Source: Owner

Figure 6 shows the number of monthly visits performed by each vehicle in the month and the percentage that each vehicle contributes to the total number of performed visits.

**Fig. 7:**
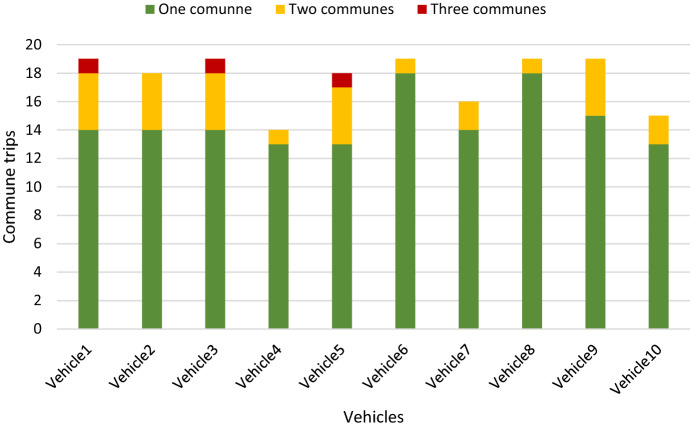
Daily trips to communes for each trip. Source: Owner

**Fig. 8:**
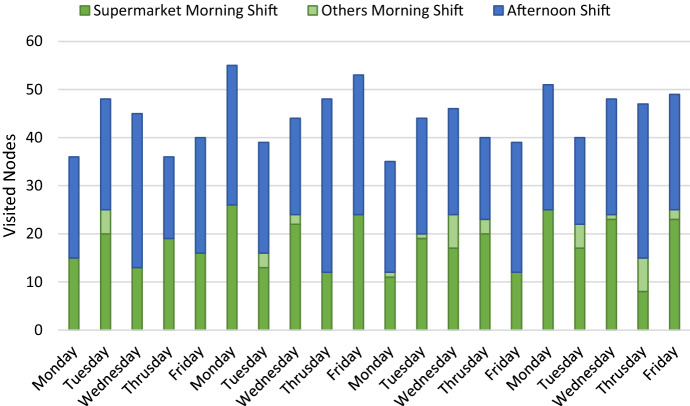
Destination nodes visited according to schedule. Source: Owner

Figure 7 shows the number of daily trips to communes performed by each vehicle. Figure 8 shows the daily visited nodes segmented by the time in which the products have been delivered.

**Fig. 9:**
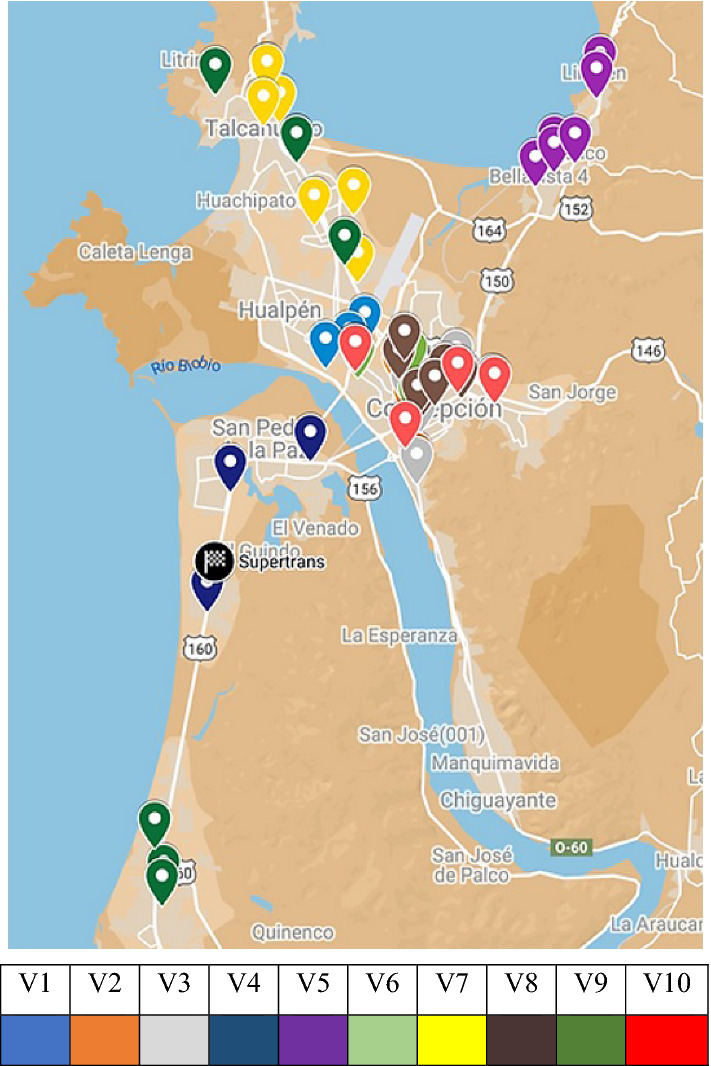
Destination node assignment, sixth business day. Source: Owner

Finally, Fig. 9 and Table 13 describe the assignment of the sixth business day of the month as an example. The elbow of the assigned destination nodes for each vehicle and the time to be visited are identified. In this case, all assigned customers with hours in the morning are supermarkets.

**Table 13:** Delivery schedule, sixth business day. Source: Owner

V1	V2	V3	V4	V5	V6	V7	V8	V9	V10
99(M)	23(M)	50(M)	2(M)	41(M)	13(M)	32(M)	220(M)	7(M)	15(M)
1(T)	30(M)	129(M)	4(M)	53(T)	17(M)	34(M)	16(M)	9(M)	18(M)
44(T)	187(M)	146(M)	3(T)	83(T)	149(M)	38(M)	19(M)	69(M)	55(M)
	70(T)	214(M)		108(T)	36(T)	22(T)	5(T)	72(T)	80(T)
	104(T)	12(T)		109(T)	122(T)	45(T)	66(T)	130(T)	
	206(T)	27(T)		194(T)	167(T)	78(T)	98(T)	180(T)	
						184(T)	209(T)		
						222(T)			

### Discussion of the results and managerial insights

The methodology executed with the study data considers a total of 122,000 variables and 53,820 constraints. Regarding the solution of the model, in 83% of the daily routes made by each vehicle, intercommunal trips are not made; that is, the vehicles visit a single commune. On the other hand, in 17% of cases, vehicles make more than one intercommunal trip, which means they visit two or three communes. Likewise, 30% of the fleet visits two communes on one day of the month; 20% visits two communes on two days of the month; 50% visits two communes on three days of the month, and 30% of the fleet visits three communes on only one day of the month.

The total number of communes visited is 209, considering 33 intercommunal trips, and the average per vehicle is 1.2 trips to communes per month. The DC made an average of 358 trips to communes and 120 intercommunal trips per month, with the model solutions being 40% and 72% better in terms of these factors than the company's current solution, respectively. The relevance of trips to communes is that they indirectly represent the distance traveled by each vehicle, influencing the delivery of products to customers.

Concerning the time parameters, note that the use of equal intercommunal times for each commune of a province can result in a vehicle that visits a commune at one end of the province to then visiting a commune at the opposite end of the same province since the exact travel times are not considered.

Vehicles have an average occupancy rate of 62%, equivalent to 375,543 kg of unoccupied capacity per month. In particular, 16 of the 20 working days of the month are not occupied, and in 12% of cases, the vehicles do not make trips. With outsourced transport services, the occupancy percentage does not influence the fixed costs of the DC. However, it does affect the variable costs since the capacity not used by the vehicles that make at least one daily route is 255,543 kg per month. As a result of the differences concerning percentages of occupation and the number of visits per vehicle, inequalities in workloads are generated. The standard deviation of the monthly trips made by the vehicles is approximately 17 trips, while the standard deviation of the monthly products delivered per vehicle is 9063 kg.

The objective of the model is not to maintain or increase vehicle occupancy. It is complex for the model to obtain a percentage of occupancy close to the vehicle's capacity while respecting reception schedules and the frequency of visits agreed upon with the customers. Real figures from the company indicate that the DC has an occupancy percentage of 64% in the distribution vehicles, which is only 2% away from the optimal solution of the proposed scheme. Due to the demands of visit frequencies and reception schedules, reducing the number of vehicles in the fleet is also not feasible. However, there are only four days of the month in which all vehicles make at least one delivery. An alternative may be to have a fleet of vehicles with less capacity, prioritizing the fulfillment of customer conditions over economies of scale. On the other hand, the design of an appropriate cost and incentive structure can help so that the differences in workloads do not affect the operations or the turnover rates of the distribution services contracted by the company. 91% of the customers visited in the morning correspond to supermarkets, while only 9% correspond to visits to destinations without time constraints.

Similarly, the average number of supermarkets visited per vehicle daily is 1.8, with only 25.3% of the total number of customers being supermarkets, and of the total demand for visits, this makeup only 38.8%. This situation happens due to the demands of time windows and the additional time penalty that affects vehicles that visit more than one supermarket daily. These figures highlight the importance of differentiating customer schedules and assigning the morning shift mainly to supermarket chains. On the other hand, the afternoon shift is longer and can be assigned to customers without specific reception hours.

The results indicate that a vehicle can visit four supermarkets during the morning shift to respect product reception schedules. The feasibility of the model is primarily linked to the total number of visits to supermarkets that must be made in the planning horizon. If an instance is required, in terms of frequency of visits and fleet size, that a vehicle must visit five supermarkets on one business day of the month, then the model would no longer be feasible for the problem. Note that disregarding travel times for calculating time windows facilitates the problem's solution; otherwise, the slack for this restriction would be considerably lower.

In short, the proposed methodology assigns the distribution vehicles in the planning horizon, respecting the factors present in a real context and minimizing intercommunal trips. This methodology establishes systematic scheduling of deliveries to the essential destination nodes of the Concepción DC. It ensures improvements in the indicators related to the management of the last mile and the fulfillment of the deadlines established with the customers. The model obtains a better fit for intercommunal trips than historical data, emphasizing the importance of distinguishing the characteristics of the destination nodes. In addition, meeting the conditions with the client through the automation of the scheduling of the order allocation process is already significant for the company's operations.

## Concluding remarks and future works

This paper proposes a new methodology for scheduling merchandise deliveries to customers based on a Real Case through an optimization model derived from meticulous data processing parameters. The general results of applying the two-phase methodology to the scheduling of the logistics process for dispatching vehicles from the distribution center are satisfactory, and the established objectives were fulfilled, achieving a total of 209 trips to communes and 33 intercommunal trips per month.

Similarly, integrating a variable transportation cost structure into the proposed model would further improve the quality of the results and provide other analysis approaches. The study methodology could be applied to the different distribution centers of the company or other distribution companies, thus improving operations globally. Even the methodology with some variations could be applied to the dispatch processes from the company's operations center to the distribution centers in the different locations, establishing a schedule in a different operational process that could benefit operations management.

Data processing with more sophisticated estimation and prediction methodologies is a future strategy. Unsupervised learning is a striking line to continue the development of this study and thus improve the quality of the optimization model inputs. Besides, developing a stochastic solution of the proposed model using Sample Average Approximation (SAA) (Escobar et al. 2013b; Mafia and Escobar, 2015; Paz et al. 2015; Rodado et al. 2017, Vélez et al. 2021) would be considered. Finally, heuristic or metaheuristic based on granular search (Escobar and Linfati 2012; Escobar et al. 2013a; Escobar et al. 2014a; Escobar et al. 2014b; Puenayán et al. 2014; Bernal et al. 2017; Bernal et al. 2018; Bernal et al. 2021; Escobar 2022 and Riaño et al. 2022) for solving large size instances could be considered.

## Supplementary Information

Below is the link to the electronic supplementary material.

Supplementary file1 (DOCX 24 KB)
