# Supplementary material for: Two-stage optimization scheme of routing scheduling from a single distribution center to multiple customers
Source: Oper Res Int J. 2023 Mar 20;23(2):25. doi: 10.1007/s12351-023-00747-z (PMC10027269; doi:10.1007/s12351-023-00747-z)
Supplement: Supplementary file 1 — Supplementary file1 (DOCX 24 KB) [file 12351_2023_747_MOESM1_ESM.docx]

**Reproducibility Report for**

**1. Metadata**

Manuscript Title: Two-stage optimization scheme of routing scheduling from a single distribution center to multiple customers

Manuscript ID (if available):

Authors: Matías Núñez-Muñoz, Rodrigo Linfati, John W. Escobar

**2. Data availability**

__X____ A. Either no data are used in this study or all data used are included in the main text or supplemental materials.

__________ B. The data used in this study is publically available at the following website* (please provide the website link).

__________ C. The data used in this study is not yet publically available but will be made publically available at the time of paper acceptance** or will be made publically available subject to an embargo period of ____ years, counting from the time of paper acceptance. If an embargo period is invoked, please explain the reason for embargo.

__________ D. The data used in this study is not and will not be made publically available due to the following reason(s). Please present the reason(s).

Note:

* One can place the data (and/or code) at his/her research website, a Github website, or any other publically accessible websites. We do expect the website holding the datasets/codesets to be stably and reliably accessible over long term. If one desires to place the data (and/or code) through IISE Transactions’ repository service, please visit IISE Transactions website, go to “Instructions for authors,” and then to “Checklist Items 9 and 10”.

** The time of paper acceptance is the time when the Accepted Manuscript Online (AMO) version of the paper is published. This time is documented for every paper in IISE Transactions on the journal’s website.

**3. Data use ethics**

__X_____ My choice in Section 2 is (A).

__________ I certify that the authors have the legitimate access to the data and that nothing in the provisions governing the use of the data prohibits the authors from using the data in this research.

**4. Computer code**^#^ **availability**

___X____ A. Either no computer code is used in this study or the settings used in software are fully described in the main text or supplemental materials.

__________ B. The computer code used in this study is publically available at the following website. (please provide the website link).

__________ C. The computer used in this study is not publically yet available but will be made publically available at the time of paper acceptance or will be made publically available subject to an embargo period of ____ years, counting from the time of paper acceptance. Please describe where to make the data publically available. If an embargo period is invoked, please explain the reason for embargo.

__________ D. The computer code used in this study is not and will not be made publically available due to the following reason(s). Please present the reason(s).

**Note:**

# If the authors run their code on a software platform (either commercial or freeware), the authors do not need to provide the software platform, but simply to provide one’s own code.

## The location for making code available and the meaning of “the time of paper acceptance” follow what is explained in Section 2.

**5. Reproducibility**

**5.1 Computer and software environment**

- Please describe the computer hardware conditions and software environment on which the authors produce the results reported in the paper.

**5.2 Workflow**

- The authors please use the following table to provide the instructions on how to reproduce the results in the figures/tables of the paper.
- The table below is supposed to include each and every figure/table in the paper that is considered as a research output or used to support the research conclusions. Hand-drawn diagrams or flowcharts are excluded from the reproducibility workflow.
- If you choose Option D above, please certify the following statement.

___X____ I certify that the authors have faithfully conducted the reproducibility tasks on their own computational devices and that the following table accurately documented the filenames used, the computational times of execution, and the outcomes.
